# 4-isopropylcyclohexanol has potential analgesic effects through the inhibition of anoctamin 1, TRPV1 and TRPA1 channel activities

**DOI:** 10.1038/srep43132

**Published:** 2017-02-22

**Authors:** Yasunori Takayama, Hidemasa Furue, Makoto Tominaga

**Affiliations:** 1 Division of Cell Signaling, Okazaki Institute for Integrative Bioscience (National Institute for Physiological Sciences), National Institutes of Natural Sciences, 5-1 Higashiyama, Myodaiji, Okazaki, Aichi, 444-8787, Japan; 2 Department of Physiological Sciences, the Graduate University for Advanced Studies, 5-1 Higashiyama, Myodaiji, Okazaki, Aichi, 444-8787, Japan; 3 Division of Neural Signaling, National Institute for Physiological Sciences, National Institutes of Natural Sciences, 5-1 Higashiyama, Myodaiji, Okazaki, Aichi, 444-8787, Japan; 4 Institute for Environmental and Gender-Specific Medicine, Juntendo University, 2-1-1 Tomioka, Urayasu, Chiba, 279-0021, Japan.

## Abstract

Interactions between calcium-activated chloride channel anoctamin 1 (ANO1) and transient receptor potential vanilloid 1 (TRPV1) enhance pain sensations in mice, suggesting that ANO1 inhibition could have analgesic effects. Here we show that menthol and the menthol analogue isopropylcyclohexane (iPr-CyH) inhibited ANO1 channels in mice. The iPr-CyH derivative 4-isopropylcyclohexanol (4-iPr-CyH-OH) inhibited mouse ANO1 currents more potently than iPr-CyH. Moreover, 4-iPr-CyH-OH inhibited the activities of TRPV1, TRP ankyrin 1 (TRPA1), TRP melastatin 8 (TRPM8) and TRPV4. Single-channel analysis revealed that 4-iPr-CyH-OH reduced TRPV1 and TRPA1 current open-times without affecting unitary amplitude or closed-time, suggesting that it affected gating rather than blocking the channel pore. The ability of 4-iPr-CyH-OH to inhibit action potential generation and reduce pain-related behaviors induced by capsaicin in mice suggests that 4-iPr-CyH-OH could have analgesic applications. Thus, 4-iPr-CyH-OH is a promising base chemical to develop novel analgesics that target ANO1 and TRP channels.

Several transient receptor potential (TRP) channels play important roles in the sensory nervous system. These channels include TRP vanilloid 1 (TRPV1), TRP ankyrin 1 (TRPA1), TRP melastatin 8 (TRPM8) and TRPV4, which are all crucial for sensing naturally occurring substances^1^,^2^,^3^,^4^. In particular, TRPV1 and TRPA1 can be activated by various nociceptive stimuli. TRPV1 is activated by capsaicin, allicin, resiniferatoxin, allyl-isothiocyanate (AITC), noxious heat, acid and tarantula toxins, whereas TRPA1 is activated by isothiocyanates (e.g., AITC), allicin, diallyl disulfide, cinnamaldehyde, methyl salicylate, alkaline, hydrogen sulfide, acrolein, ozone, ammonia, formalin, disulfiram and glibenclamide^5^,^6^,^7^,^8^,^9^,^10^,^11^,^12^,^13^,^14^,^15^. Although TRP channels were cloned some time ago and their inhibition would be a clear strategy for pain reduction, no clinical channel antagonists are available, in part because specifically antagonizing these channels can have potentially serious side effects. For instance, a TRPV1 antagonist, AMG 517, induces hyperthermia reaching 38 °C for 12 hr after administration in human (ref. 16). Recent reports showed that the calcium-activated chloride channel anoctamin 1 (ANO1, also known as TMEM16A), which is co-expressed with TRPV1 in dorsal root ganglia (DRG) neurons, is activated by calcium released from the endoplasmic reticulum and is sensitive to rapid noxious heating, thus making ANO1 another key factor in nociception^17^,^18^,^19^,^20^,^21^. Mice having conditional knock out or knock down of ANO1 expression in DRG neurons showed a reduction in pain-related behaviors induced by heat or bradykinin^20^,^21^. Local increases in intracellular calcium are important for ANO1 activation and downstream events, such as the functional and physical interaction between ANO1 and inositol trisphosphate receptors following activation of Gq-protein coupled receptors^18^,^22^. However, global increases in calcium are also likely to be involved in ANO1 activation and mediate its interactions^22^,^23^. In addition, we recently reported that local calcium influx through the membrane ion channel TRPV1 activated ANO1 within a physical complex, and this activation was followed by pain enhancement through additional depolarization resulting from TRPV1/ANO1 interactions^24^. We also showed that a known ANO1 inhibitor reduced TRPV1-mediated pain-related behaviors in mice^24^. These results suggest that the apparent inward currents evoked by capsaicin in mouse sensory neurons have two components: TRPV1-mediated cationic inward currents and ANO1-mediated outward chloride currents, both of which contribute to the depolarization needed to generate action potentials. Thus, the development of ANO1 antagonists could lead to promising new methods for reducing pain sensations. In this study, we found that menthol inhibited ANO1 currents evoked by calcium. Menthol has long been used to treat pain^25^,^26^, and is a frequent additive in both cosmetics and processed foods for its cooling properties. Menthol is also an agonist for TRPM8, which is expressed in various tissues, including primary sensory neurons^3^,^27^. Furthermore, TRPM8-expressing afferent neurons were reported to inhibit a neural network involved in heat and/or mechanical nociception in neuropathic conditions^28^,^29^. Although menthol induces a cool sensation through excitation of TRPM8-expressing DRG neurons, the pharmacological effects of menthol are not specific to TRPM8. For instance, menthol also activates TRPA1 and TRPV3 (refs 30 and 31). On the other hand, menthol inhibits AITC-induced activation of human TRPA1 through a serine and a threonine residue in TRPA1 transmembrane helix 5 (ref. 30). Menthol also inhibits human TRPV1 activation by capsaicin^32^, whereas rat TRPV1 was not affected^33^. The results of these previous studies explain both the analgesic and proalgesic effects of menthol. Here, we attempted to find the basic chemical structure for menthol-mediated ANO1 inhibition and identified a novel analgesic compound, 4-isopropylcyclohexanol (4-iPr-CyH-OH), which acts on ANO1, TRPV1, TRPA1, TRPM8 and TRPV4.

## Results

### Pharmacological interactions between TRP channels and ANO1

**Figure 1:**
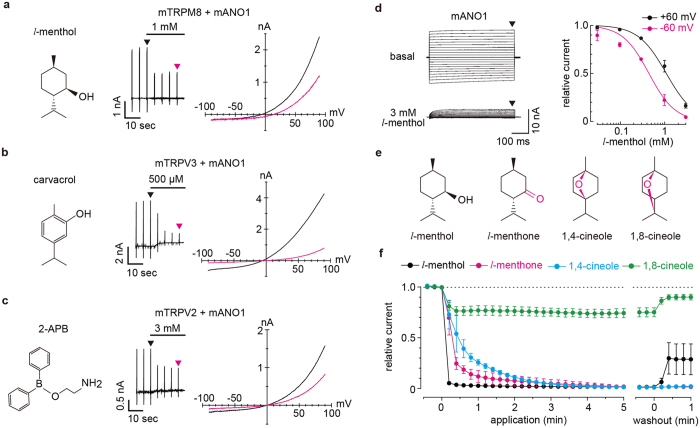
Inhibition of mANO1 currents by menthol. (**a**–**c**) Chemical structures of *l*-menthol, carvacrol and 2-APB (left), typical traces (middle) and I-V curves (right) of chloride currents in HEK293T cells expressing mANO1 with mTRPM8 (**a**), mTRPV3 (**b**) and mTRPV2 (**c**). Holding potentials were −60 mV with ramp-pulses (−100~+100 mV, 300 ms). Basal ANO1 currents were induced by 100 nM intracellular free calcium. Arrowheads indicate points of the I-V curve. (**d**) Representative traces of mANO1 currents in HEK293T cells induced by 500 nM intracellular free calcium in the absence or presence of 3 mM *l*-menthol (left). Holding potential was 0 mV and 500 ms step pulses (each 10 mV) were applied every 1 sec. Arrowheads indicate the point for relative current calculation in the dose-response curve of *l*-menthol (right). EC_50_ values were 441 μM and 1.07 mM at −60 (magenta) and +60 mV (black) membrane potentials, respectively. (**e**) Chemical structures of *l*-menthol and its analogues. (**f**) Time course of changes in relative mANO1 currents induced by 500 nM intracellular free calcium in the presence of the four chemicals (3 mM).

We previously reported functional interactions between TRP channels (TRPV4, TRPV1 and TRPA1) and ANO1 in mice^24^,^34^. In screens for other interactions of TRP channels with ANO1 in HEK293T cells, we found that 1 mM *l*-menthol, 500 μM carvacrol and 3 mM 2-aminoethoxydiphenylborane (2-APB) inhibited mouse ANO1 (mANO1) currents induced by 100 nM free calcium (Fig. 1a–c). We focused on the mechanism of menthol-induced inhibition because we can compare the inhibitory effects with different menthol analogues. mANO1 activation reached saturation with 500 nM free intracellular calcium (Supplementary Fig. 1), although the values varied among previous reports^18^,^35^. Given these discrepancies, we first characterized the effects of menthol on mANO1 at 500 nM calcium. We found that 3 mM *l*-menthol, which also has agonist effects on TRPM8, almost completely inhibited mANO1 currents with half-maximal inhibitory concentrations (IC_50_) of 441 μM and 1.07 mM at −60 mV and +60 mV, respectively (Fig. 1d).

To identify chemicals that had a greater capacity to inhibit ANO1, we examined several menthol analogues in assays involving the application of voltage-step pulses (from 0 mV to −60 mV for 500 ms) every 12 sec over 5 min. The mANO1 currents were almost completely inhibited by 3 mM *l*-menthol, *l*-menthone or 1,4-cineole, but not by 1,8-cineole (Fig. 1e,f). The *l*-menthol effect was the most rapid among the 3 compounds, whereas *l*-menthone and 1,4-cineole were difficult to washout. We inferred the active entity of the smallest structure in menthol based on these differences between the analogues.

### Identification of the core structure for inhibition of ANO1 by menthol

**Figure 2:**
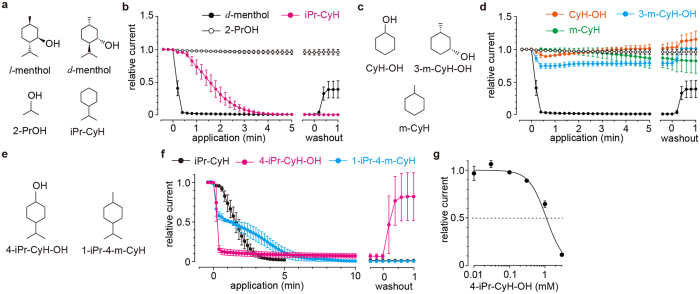
Identification of the core structure involved in menthol inhibition of mANO1 currents. (**a**) Chemical structures of *l*-menthol, *d*-menthol, 2-propanol (2-PrOH) and isopropylcyclohexane (iPr-CyH). (**b**) Time courses of the relative mANO1 currents affected by the indicated compounds at 3 mM. (**c**) Chemical structures of cyclohexanol (CyH-OH), 3-methylcyclohexanol (3-m-CyH-OH) and methylcyclohexane (m-CyH). (**d**) Time courses of the relative mANO1 currents affected by the indicated compounds at 3 mM. (**e**) Chemical structures of 4-isopropylcyclohexanol (4-iPr-CyH-OH) and 1-isopropyl-4-methyl-cyclohexane (1-iPr-4-m-CyH). (**f**) Time courses of the relative currents affected by 3 mM 4-iPr-CyH-OH (magenta) and 1-iPr-4-m-CyH (cyan). Black indicates the time course of the iPr-CyH effect in **b**. (**g**) Dose-response curve for 4-iPr-CyH-OH-induced inhibition of mANO1 currents. The IC_50_ was 1.09 mM.

We next attempted to identify the core structure in menthol that promotes ANO1 inhibition. Based on the structural differences between *l*-menthol, *l*-menthone, 1,4-cineole and 1,8-cineole, we focused on the isopropyl group, since the effect of 1,8-cineole was smaller than that of the other three compounds (Fig. 1f). We also examined the effects of *d*-menthol because the mechanism of TRPM8 activation by *l*- and *d*-menthol were reportedly different^36^. However, we saw no difference in the effects of the two isomers on mANO1 (Figs 1f and 2b). We observed that isopropylcyclohexane (iPr-CyH) strongly inhibited mANO1 currents to a similar degree as that seen for *d*-menthol although 2-propanol (2-PrOH) had no effect (Fig. 2a,b). In contrast, other functional groups, including cyclohexanol (CyH-OH), 3-methylcyclohexanol (3-m-CyH-OH) and methylcyclohexane (m-CyH), showed little effect on mANO1 activity (Fig. 2c,d). These results suggested that iPr-CyH is a key structure that confers inhibitory action toward mANO1.

Because the inhibitory effect of iPr-CyH was slower than that of menthol, we compared the hydrophilicity of the menthol analogues. The estimated octanol/water partition coefficient (K_OW_) for iPr-CyH is higher than that of menthol (K_OW_ = 4.54 and 3.14 for iPr-CyH and menthol, respectively, at pH 7.40) according to ACD/Labs (http://www.acdlabs.com/products/percepta/predictors.php). Consistent with this notion, mANO1 currents were rapidly inhibited by 4-iPr-CyH-OH that has a low K_OW_ value (2.77) and an IC_50_ of 1.09 mM at −60 mV (Fig. 2e–g). In contrast, 1-isopropyl-4-methylcyclohexane (1-iPr-4-m-CyH, K_OW_ = 5.09) showed two inhibitory phases (rapid and slow), although it too completely inhibited ANO1 currents (Fig. 2f). These results suggested two potentially important chemical determinants for mANO1 inhibition: the presence of an iPr-CyH group and its hydrophilicity, and that a hydroxyl group is not critically involved.

### Effects of 4-iPr-CyH-OH on mouse TRPV1 and TRPA1

**Figure 3:**
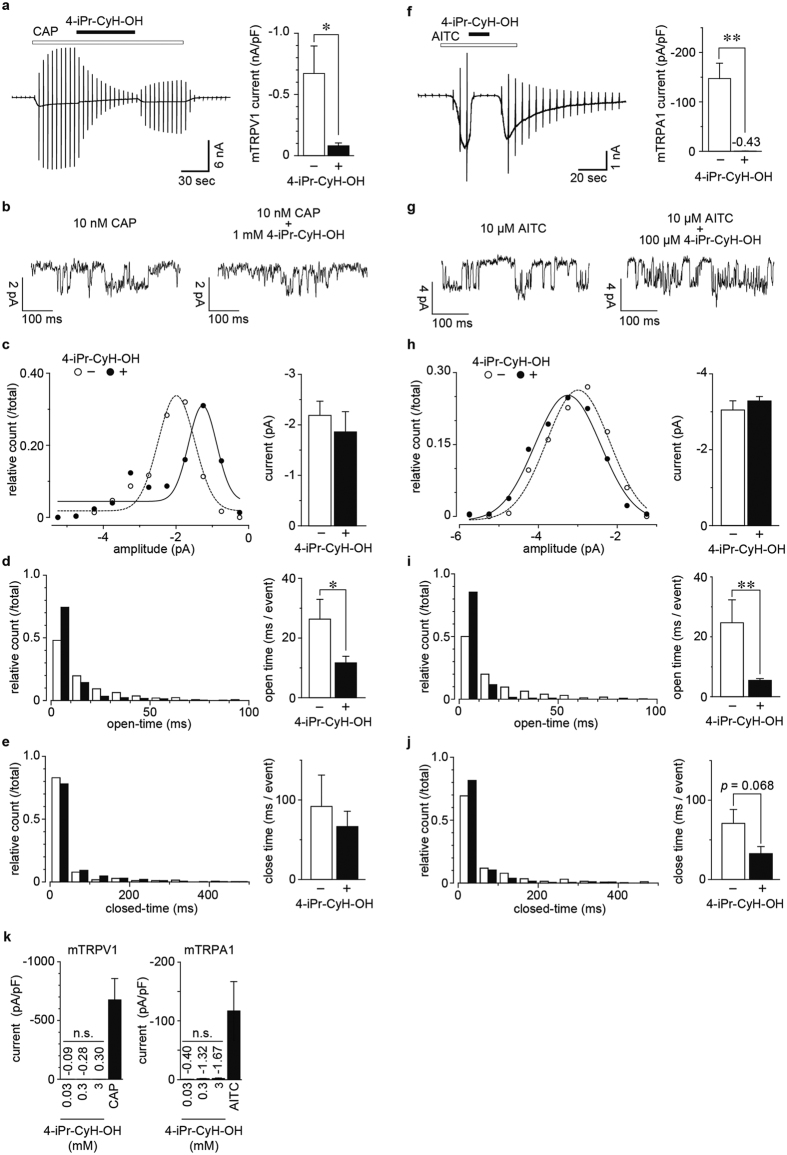
4-iPr-CyH-OH effects on mouse TRPV1 and TRPA1. (**a**) A typical trace and amplitudes of capsaicin (100 nM)-activated mTRPV1 currents inhibited by 3 mM 4-iPr-CyH-OH in HEK293T cells. Holding potentials were 0 mV with ramp-pulses (−100~+100 mV, 300 ms). (**b**–**f**) Single channel analyses of mTRPV1 currents induced by 10 nM capsaicin (CAP) with or without 1 mM 4-iPr-CyH-OH. Each figure indicates the typical traces (**b**), amplitude (**c**), open-time (**d**) and closed-time (**e**). Each bar graph indicates the averaged value. (**f**) A typical trace and amplitudes of allyl isothiocyanate (AITC, 300 μM)-activated mTRPA1 currents inhibited by 3 mM 4-iPr-CyH-OH in HEK293T cells. Holding potentials were −60 mV with ramp-pulses (−100~+100 mV, 300 ms). (**g**–**j**) Single channel analyses in mTRPA1 currents induced by 10 μM AITC with or without 100 μM 4-iPr-CyH-OH. *p < 0.05, **p < 0.01, Student’s t-test. (**k**) Averaged amplitudes of mouse TRPV1 (mTRPV1, left) and TRPA1 (mTRPA1, right) in 4-iPr-CyH-OH applications (0.03, 0.3 and 3 mM). The concentration of capsaicin (CAP) and AITC was 1 μM and 300 μM, respectively. n.s., not significant, Bonferroni test.

We recently described the inhibition of human TRPV1 (hTRPV1) by menthol, a mechanism that might also be related to menthol-induced anti-nociception^32^, and we found that menthol had similar effects on mTRPV1 (Supplementary Fig. 2). Therefore, we investigated the effects of 4-iPr-CyH-OH on mTRPV1. Treatment of HEK293T cells expressing mTRPV1 with 3 mM 4-iPr-CyH-OH inhibited mTRPV1-mediated currents induced by 100 nM capsaicin (Fig. 3a), and the currents recovered slightly after 4-iPr-CyH-OH washout. To investigate this inhibition mechanism, we analyzed single-channel currents in inside-out patch-clamp recordings at a −60 mV pipette holding potential (membrane potential was +60 mV). In these assays we detected 50 single channel events in the stable phase of channel activation and calculated the average values for amplitude, open-time and closed-time for each recording. The average single-channel open-time decreased, but neither single channel conductance nor closed-time was changed significantly in the presence of 4-iPr-CyH-OH (Fig. 3b–e). These results indicated that 4-iPr-CyH-OH affects the gating of ion channels, but does not directly block the channel pore because the channel conductance was not affected by 4-iPr-CyH-OH. Meanwhile, mTRPA1 currents induced by 300 μM AITC were also inhibited by 4-iPr-CyH-OH (Fig. 3f), and its effects on single channel properties were similar to those for mTRPV1 wherein only the open-time was affected (Fig. 3g–j). Thus, the inhibitory mechanism of 4-iPr-CyH-OH on both mTRPV1 and mTRPA1 appears to involve modification of channel gating. Since some TRP channel agonists exhibit bimodal effects, as was seen for menthol activation of TRPA1 and inhibition of AITC-mediated TRPA1 activation (ref. 37), we also investigated the agonistic effects of 4-iPr-CyH-OH on mTRPV1 and mTRPA1 in whole-cell patch-clamp recordings. We calculated the current densities at −60 mV, and observed no current activation of the two channels by 4-iPr-CyH-OH (0.03, 0.3 and 3 mM), although channel activity was induced by their respective agonists (Fig. 3k). Together these results show that 4-iPr-CyH-OH had antagonistic effects on both mTRPV1 and mTRPA1 channels expressed in HEK293T cells.

### Inhibition of pain sensation by 4-iPr-CyH-OH

**Figure 4:**
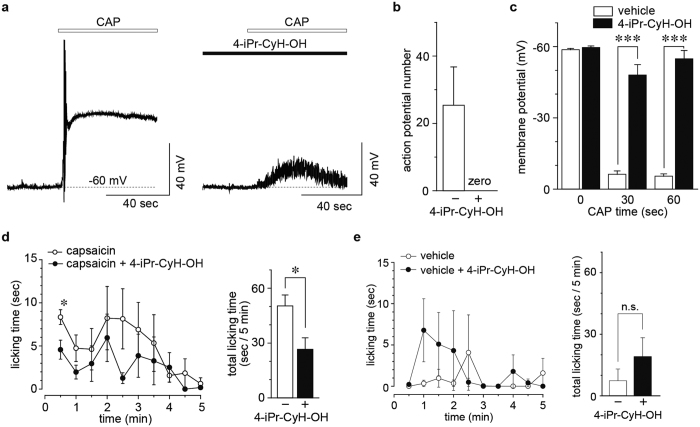
Analgesic effect of 4-iPr-CyH-OH. (**a**) Typical traces of capsaicin (1 μM)-induced action potentials in small DRG neurons with or without 3 mM 4-iPr-CyH-OH. Resting potentials were maintained at −60 mV. (**b**,**c**) Averaged numbers of action potentials induced by capsaicin (**b**) and averaged values of membrane potentials at 0, 30 and 60 seconds after capsaicin application (**c**) with or without 4-iPr-CyH-OH. ***p < 0.001, Student’s t-test. (**d**) Occurrence of pain-related behaviors during 30 sec intervals (left) and total occurrence over 5 min (right) in mice administered with 300 μM capsaicin with or without 3 mM 4-iPr-CyH-OH to the hind paw. (**e**) Occurrence of pain-related behaviors during 30 sec intervals (left) and the total occurrence over a 5 min period (right) in WT mice administered with 3 mM 4-iPr-CyH-OH or vehicle to the hind paw. *p < 0.05, n.s., not significant, Mann-Whitney U test.

The findings that 4-iPr-CyH-OH inhibited mTRPV1 and mTRPA1 activation without agonistic effects on these channels suggest that 4-iPr-CyH-OH could reduce pain sensation by inhibiting multiple TRP channels involved in pain generation. This effect would presumably not be accompanied by the irritation that typically results from TRPV1 or TRPA1 activation in mice. Therefore, we investigated whether 4-iPr-CyH-OH inhibits action potentials evoked by 1 μM capsaicin in isolated small DRG neurons. We previously showed that the selective ANO1 inhibitor T16Ainh-A01 reduced action potential generation promoted by TRPV1-ANO1 interactions, although capsaicin-induced depolarization still occurred^24^. Here, 3 mM 4-iPr-CyH-OH not only strongly inhibited neuronal membrane depolarization, but also eliminated action potential generation (Fig. 4a–c). This effect of 4-iPr-CyH-OH is probably due to its actions on both TRPV1 and ANO1, which again supports the possibility that 4-iPr-CyH-OH could have analgesic properties. Although capsaicin (1 μM) caused some depolarization during a 60 sec application in the presence of 4-iPr-CyH-OH (Fig. 4a), the depolarization was drastically inhibited compared to that seen in the absence of 4-iPr-CyH-OH (Fig. 4c). Therefore, we performed a pain-related behavior test in mice that were subcutaneously injected with 300 μM capsaicin with or without 3 mM 4-iPr-CyH-OH. Treatment with 4-iPr-CyH-OH indeed significantly reduced pain-related behaviors *in vivo* (Fig. 4d), whereas 4-iPr-CyH-OH alone did not promote significant increases in pain-related behaviors (Fig. 4e). These results suggested that 4-iPr-CyH-OH could be a candidate analgesic.

### Effects of 4-iPr-CyH-OH on human ANO1, TRPV1 and TRPA1

**Figure 5:**
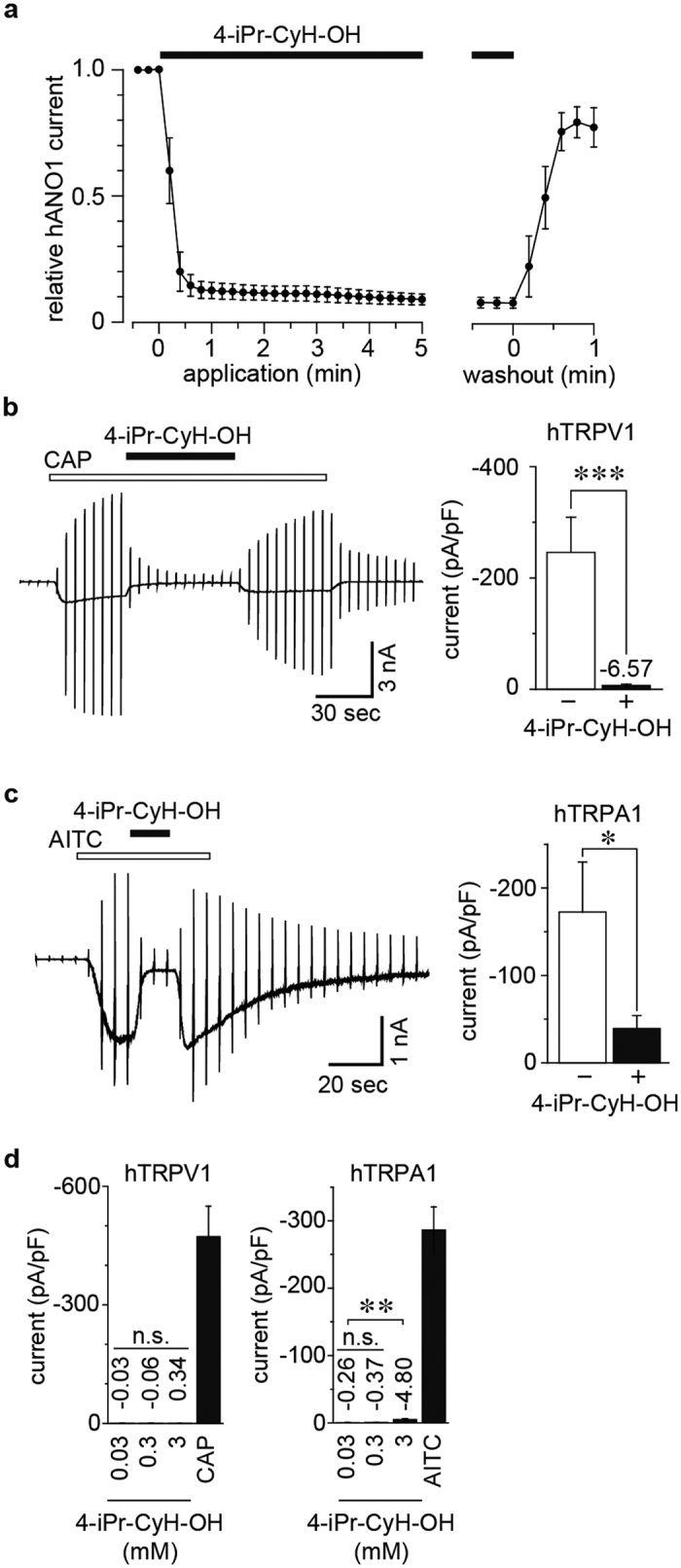
Effects of 4-iPr-CyH-OH on human ion channels. (**a**) Time course of relative human ANO1 (hANO1) currents in the presence of 3 mM 4-iPr-CyH-OH. (**b**) Typical trace (left) and averaged amplitudes (right) of capsaicin (100 nM)-activated hTRPV1 currents inhibited by 3 mM 4-iPr-CyH-OH in HEK293T cells. Holding potentials were 0 mV with ramp-pulses (−100~+100 mV, 300 ms). (**c**) Typical trace (left) and averaged amplitudes (right) of AITC (300 μM)-activated hTRPA1 currents inhibited by 3 mM 4-iPr-CyH-OH in HEK293T cells. Holding potentials were −60 mV with ramp-pulses (−100~+100 mV, 300 ms). *p < 0.05, ***p < 0.001, Student’s t-test. (**d**) Averaged amplitudes of human TRPV1 (hTRPV1, left) and TRPA1 (hTRPA1, right) in 4-iPr-CyH-OH applications (0.03, 0.3 and 3 mM). The concentration of capsaicin (CAP) and AITC was 1 μM and 300 μM, respectively. **p < 0.01, n.s., not significant, Bonferroni test.

The effects of individual antagonists could differ between mouse and human channels. For example, caffeine activates mTRPA1 but inhibits hTRPA1 (ref. 38). Moreover, comparisons between mouse and human channels would be important when considering potential clinical applications of compounds. Therefore, we investigated the effects of 4-iPr-CyH-OH on human ANO1 (hANO1), hTRPV1 and hTRPA1. Similar to mANO1 (Fig. 2f), hANO1 currents induced by 500 nM intracellular free calcium were inhibited by 3 mM 4-iPr-CyH-OH (Fig. 5a). Both capsaicin (100 nM)-induced hTRPV1 currents and AITC (300 μM)-induced hTRPA1 currents were also inhibited by 3 mM 4-iPr-CyH-OH (Fig. 5b,c). Furthermore, as with mouse clones (Fig. 3k), 4-iPr-CyH-OH did not activate hTRPV1 (Fig. 5d), yet did slightly but significantly activate hTRPA1 (Fig. 5d). This incomplete inhibition of hTRPA1 by 4-iPr-CyH-OH (Fig. 5c) could be in part related to the small activation of hTRPA1 induced by 4-iPr-CyH-OH.

### Effects of 4-iPr-CyH-OH on TRPM8 and TRPV4

**Figure 6:**
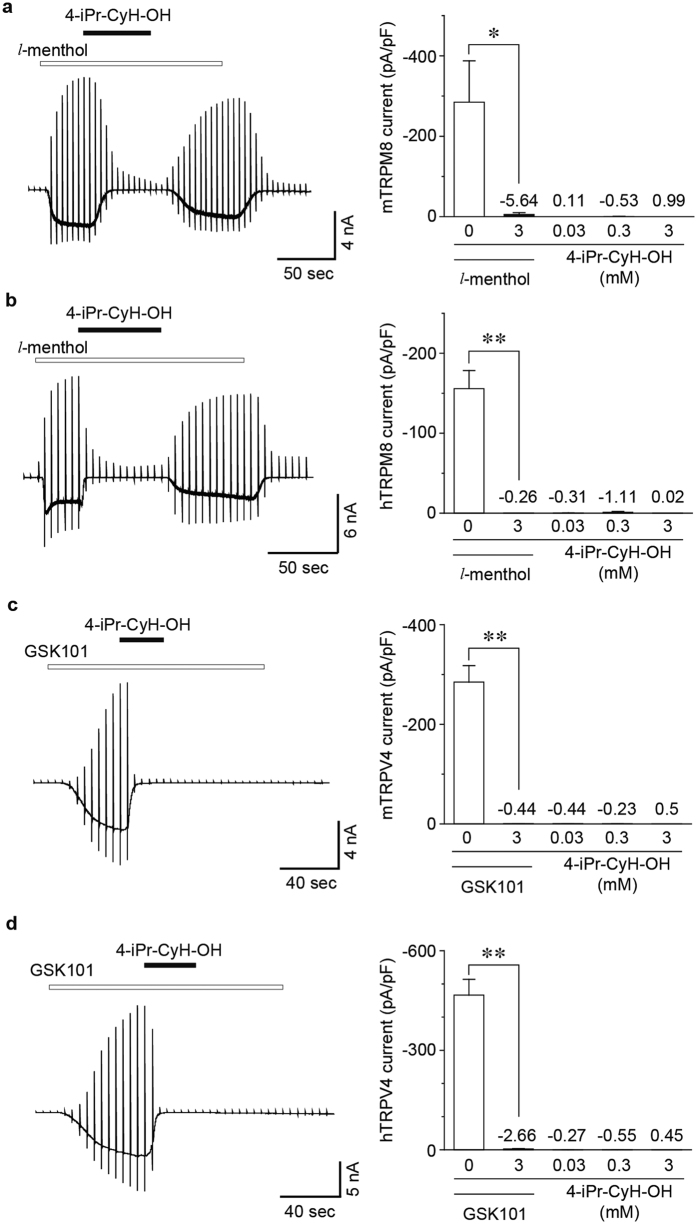
Effects of 4-iPr-CyH-OH on TRPM8 and TRPV4. (**a,b**) Typical traces (left) and the averaged amplitudes (right) of *l*-menthol (500 μM)-activated currents of mouse (**a**) and human (**b**) TRPM8, which were both inhibited by 3 mM 4-iPr-CyH-OH in HEK293T cells. Neither mouse nor human TRPM8 was activated by 4-iPr-CyH-OH (0.03, 0.3 and 3 mM). (**c,d**) Typical traces (left) and the averaged amplitudes (right) of GSK1016790A (GSK101, 300 nM)-activated currents of mouse (**c**) and human (**d**) TRPV4, both of which were irreversibly inhibited by 3 mM 4-iPr-CyH-OH in HEK293T cells. Neither mouse nor human TRPV4 was activated by 4-iPr-CyH-OH (0.03, 0.3 and 3 mM). Holding potentials were −60 mV with ramp-pulses (−100~+100 mV, 300 ms). *p < 0.05, **p < 0.01, Student’s t-test.

TRPM8-expressing DRG neurons were reported to show inhibition of pain sensation pathway in the spinal cord depending on TRPV1 activation^29^, which could explain the observed menthol-induced anti-nociception. Furthermore, TRPV4 in sensory neurons is also known to be involved in nociception^39^,^40^,^41^,^42^,^43^,^44^. Therefore, we investigated the effects of 4-iPr-CyH-OH on mTRPM8, hTRPM8, mTRPV4 and hTRPV4. Interestingly, both *l*-menthol (500 μM)-induced TRPM8 currents and GSK1016790A (GSK101, 300 nM)-induced TRPV4 currents were inhibited by concomitant administration of 3 mM 4-iPr-CyH-OH, whereas inhibition of TRPV4 currents by 4-iPr-CyH-OH was not recovered after washout (Fig. 6). Additionally, TRPM8 and TRPV4 were not activated by 4-iPr-CyH-OH.

## Discussion

In this study we found that menthol inhibited ANO1 channel activity. We also identified a novel analgesic mechanism for the menthol analogue 4-iPr-CyH-OH, which inhibited TRPV1, TRPA1, TRPM8, TRPV4 and ANO1, as well as mitigated capsaicin-induced pain-related behaviors in mice. Although our results suggest that 4-iPr-CyH-OH would have analgesic properties, they could be highly non-specific given that ANO1 is expressed in most epithelial cells. Nonetheless, 4-iPr-CyH-OH should provide a good foundation on which to develop novel analgesic agents, particularly because it could inhibit four major ion channels involved in nociception in primary afferent neurons. Moreover, the absence of an agonist effect for 4-iPr-CyH-OH on these five channels may reduce side effects and dosages. This possibility is supported by recent reports showing that the dual inhibitor of TRPV4 and TRPA1, compound 16-8, more effectively reduces TRPV4-related nociception in trigeminal ganglia compared with a single administration of the TRPV4 specific antagonist, GSK205 (ref. 45).

As described above, systemic administration of ANO1 inhibitors might be unsuitable for analgesic purposes because ANO1 is expressed in most epithelial cells and is involved in various functions, including smooth muscle contraction^46^, mucin secretion^47^,^48^ and insulin release^49^,^50^. Despite its weak activation of hTRPA1 (Fig. 5d), 4-iPr-CyH-OH likely would not induce TRPA1-related pain sensation in humans at therapeutic doses. In addition, chemical modifications to 4-iPr-CyH-OH could be made that would reduce its effects on hTRPA1. Moreover, the ability of 4-iPr-CyH-OH to inhibit mouse and human ANO1, TRPV1, TRPA1 and TRPV4 channels is a promising feature for clinical applications because many TRP channel agonists are known to act promiscuously on different TRP channels.

The inside/out single-channel analyses revealed that 4-iPr-CyH-OH reduced capsaicin-induced TRPV1 and TRPA1 open-time induced by capsaicin and AITC, respectively, without affecting unitary conductance or closed-time. This result suggests that 4-iPr-CyH-OH affects channel gating. However, the tendency towards reductions in unitary amplitude in the presence of 4-iPr-CyH-OH and rapid recovery of TRPA1 currents by 4-iPr-CyH-OH washout in whole-cell recordings indicates that pore blocking of TRPV1 and TRPA1 may also be involved in 4-iPr-CyH-OH-mediated inhibition to some extent. The finding that 4-iPr-CyH-OH inhibited both cation (TRPV1, TRPA1, TRPM8 and TRPV4) and anion (ANO1) channels suggests the existence of a common structure that controls gating in both cation and anion channels, at least for TRPV1, TRPA1, TRPM8, TRPV4 and ANO1. However, we could not obtain single-channel recordings for ANO1 channels. Although the structures of TRPV1, TRPA1 and TMEM16 homologues were determined by cryo-EM single particle analysis or crystallography, they do not yet have sufficient resolution to allow us to define a structural basis for 4-iPr-CyH-OH inhibition of the three channels^51^. As the resolution improves, however, 4-iPr-CyH-OH modeling might be performed similarly to the structure-based approach used to design opioids with fewer side effects^52^. In addition, given that 4-iPr-CyH-OH inhibited the five different channels, future studies should characterize the structural basis for the inhibition, which could enhance our understanding of mechanisms involved in channel gating.

In conclusion, we propose that 4-iPr-CyH-OH is a key component for designing optimal analgesia agents that inhibit multiple ion channels involved in peripheral nociception.

## Materials and Methods

### Animals

All mouse experiments were conducted with six- to eight-week-old C57BL/6NCr mice. All procedures involving the care and use of animals were approved by the institutional Animal Care and Use Committee of the National Institute for Physiological Sciences (#16A074) and were carried out in accordance with National Institutes of Health *Guide for the care and use of laboratory animals* (NIH publication No. 85-23. Revised, 1985).

### Chemicals

*l*- and *d*-menthol, capsaicin, 1,4-cineole, 1,8-cineole, isopropylcyclohexane (iPr-CyH) and cyclohexanol (CyH-OH) were purchased from Sigma-Aldrich (United States). 4-isopropylcyclohexanol (4-iPr-CyH-OH), methylcyclohexane (m-CyH), cis-3-methylcyclohexanol (3-m-CyH-OH) and cis-1-isopropyl-4-methylcyclohexane (1-m-4-iPr-CyH) were purchased from Tokyo Chemical Industry (Japan). Allyl isothiocyanate (AITC) and 2-propanol (2-PrOH) were purchased from Wako (Japan). The estimated values for the octanol/water partition coefficients were obtained from an online database (ChemSpider).

### Whole-cell voltage-clamp recordings

HEK293T cells were cultured in DMEM (high glucose) with L-glutamine and phenol red (Wako, Japan) containing 10% FBS (lot# S06537S1560, Biowest, France), penicillin/streptomycin (1:200, Life Technologies, United States) and GlutaMax (1:100, Life Technologies) at 37 °C in a humidified chamber containing 5% CO_2_. The cells were transfected with 0.5 μg cDNA carrying mouse Ano1 (a generous gift from Dr. U. Oh), human ANO1_abcd (a generous gift from Dr. L. Galietta), mouse Trpm8, mouse Trpa1, human TRPA1 (generous gifts from Dr. A. Patapoutian), mouse Trpv1 (a generous gift from Dr. M. Caterina), mouse TRPV4 (cloned by RIKEN), human TRPV1 (a generous gift from Dr. Y. Mori), human TRPV4 (a generous gift from Dr. W. Liedtke) or human TRPM8 (cloned by our lab) using Lipofectamine (Invitrogen, United States). The cells were used 24 to 30 h after transfection. The bath solution contained 140 mM NaCl or N-methyl-D-glucamine (NMDG)-Cl for TRP and ANO1 channels, respectively), 1 mM MgCl_2_, 5 mM ethylene glycol tetraacetic acid (EGTA) or 2 mM CaCl_2_ (for TRP channels or ANO1, respectively), 10 mM glucose and 10 mM HEPES, pH 7.40, adjusted with NaOH or NMDG. The pipette solution contained 140 mM NMDG-Cl, 1 mM MgCl_2_, 5 mM 1,2-bis(o-aminophenoxy)ethane -N,N,N′,N′-tetraacetic acid (BAPTA), and 10 mM HEPES, pH 7.30, adjusted with NMDG. The free calcium concentration in the pipette solution was calculated using the MAXC program (Stanford University). Currents were recorded using an Axopatch 200B amplifier (Molecular Devices, United States), filtered at 5 kHz with a low-pass filter, and digitized with Digidata 1440 A (Axon Instruments, United States). Data were acquired with pCLAMP 10 (Axon Instruments, United States). Between 3 and 9 trials were performed for each experiment.

### Single channel voltage-clamp recordings

HEK293T cells were transfected with 0.1 to 0.3 μg cDNA carrying mouse Trpv1 or mouse Trpa1 using Lipofectamine. The cells were used 20 to 24 h after transfection. Recordings were performed in an inside-out mode at −60 mV holding potentials. Bath and pipette solutions contained 140 mM NaCl, 5 mM EGTA, 10 mM glucose and 10 mM HEPES, pH 7.40, adjusted with NaOH. Fifty events were continuously detected during the stable phase of channel activation induced by capsaicin (50 nM) or AITC (10 μM). Single channel amplitude, opening times and closing times were measured using Clampfit 10. One mM and 100 μM 4-iPr-CyH-OH were used for experiments involving TRPV1 and TRPA1, respectively. Gauss fittings were performed with Origin Pro 2015 J (OriginLab, United States). Between 6 and 8 trials were performed for each experiment.

### Whole-cell current-clamp recordings in DRG neurons

Data were collected from small DRG neurons (<24 μm) isolated by the same protocol as previously reported^24^. The bath solution contained 140 mM NaCl, 5 mM KCl, 1 mM MgCl_2_, 2 mM CaCl_2_, 10 mM glucose and 10 mM HEPES, pH 7.40, adjusted with NaOH. The pipette solution contained 67 mM KCl, 65 mM K-gluconate, 1 mM MgCl_2_, 5 mM EGTA, 4 mM ATP-Mg, 1 mM GTP-Na_2_ and 10 mM HEPES, pH 7.30, adjusted with KOH. Free calcium was maintained at 100 nM. Basal voltages were maintained around −50 mV by current injection. Five trials were performed for each experiment.

### Pain-related behavioral tests

Behavioral tests were performed using the same protocol as previously reported^24^. Briefly, 10 μL of 300 μM capsaicin with or without 3 mM iPr-CyH-OH diluted in saline containing 0.3% ethanol and 3% DMSO was used to subcutaneously inject the top of mouse hind paws using a 25 μl Hamilton syringe fitted with a 30 gauge needle. Licking behavior was recorded using a digital camera (P6000, Nikon, Japan). Between 5 and 8 trials were performed for each experiment.

### Statistical analyses

Statistical analyses were performed with Origin Pro 2015 J (OriginLab, United States). Student’s T-test and Bonferroni analyses were performed for comparisons between groups. Behavioral tests were typically analyzed with the Mann-Whitney U test. Values of p < 0.05 were accepted as statistically significant differences.

## Additional Information

**How to cite this article:** Takayama, Y. *et al*. 4-isopropylcyclohexanol has potential analgesic effects through inhibition of anoctamin 1, TRPV1 and TRPA1 channel activities. *Sci. Rep.* **7**, 43132; doi: 10.1038/srep43132 (2017).

**Publisher's note:** Springer Nature remains neutral with regard to jurisdictional claims in published maps and institutional affiliations.

## Supplementary Material

Supplementary Information

## References

[b1] Caterina M. J., Schumacher M. A., Tominaga M., Rosen T. A., Levine J. D. & Julius D. The capsaicin receptor: a heat-activated ion channel in the pain pathway. Nature 389, 816–824 (1997). 9349813 10.1038/39807

[b2] Jordt S. E. . Mustard oils and cannabinoids excite sensory nerve fibres through the TRP channel ANKTM1. Nature 427, 260–265 (2004). 14712238 10.1038/nature02282

[b3] McKemy D. D., Neuhausser W. M. & Julius D. Identification of a cold receptor reveals a general role for TRP channels in thermosensation. Nature 416, 52–58 (2002). 11882888 10.1038/nature719

[b4] White J. P., Cibelli M., Urban L., Nilius B., McGeown J. G. & Nagy I. TRPV4: Molecular Conductor of a Diverse Orchestra. Physiol Rev 96, 911–973 (2016). 27252279 10.1152/physrev.00016.2015

[b5] Salazar H. . A single N-terminal cysteine in TRPV1 determines activation by pungent compounds from onion and garlic. Nat Neurosci 11, 255–261 (2008). 18297068 10.1038/nn2056 PMC4370189

[b6] Ohta T., Imagawa T. & Ito S. Novel agonistic action of mustard oil on recombinant and endogenous porcine transient receptor potential V1 (pTRPV1) channels. Biochem Pharmacol 73, 1646–1656 (2007). 17328867 10.1016/j.bcp.2007.01.029

[b7] Everaerts W. . The capsaicin receptor TRPV1 is a crucial mediator of the noxious effects of mustard oil. Curr Biol 21, 316–321 (2011). 21315593 10.1016/j.cub.2011.01.031

[b8] Gees M. . Mechanisms of transient receptor potential vanilloid 1 activation and sensitization by allyl isothiocyanate. Mol Pharmacol 84, 325–334 (2013). 23757176 10.1124/mol.113.085548

[b9] Alpizar Y. A. . Allyl isothiocyanate sensitizes TRPV1 to heat stimulation. Pflugers Arch 466, 507–515 (2014). 23955021 10.1007/s00424-013-1334-9

[b10] Talavera K., Nilius B. & Voets T. Neuronal TRP channels: thermometers, pathfinders and life-savers. Trends Neurosci 31, 287–295 (2008). 18471901 10.1016/j.tins.2008.03.002

[b11] Smith H. S. Hydrogen sulfide’s involvement in modulating nociception. Pain Physician 12, 901–910 (2009). 19787017

[b12] Julius D. TRP channels and pain. Annu Rev Cell Dev Biol 29, 355–384 (2013). 24099085 10.1146/annurev-cellbio-101011-155833

[b13] Nilius B., Appendino G. & Owsianik G. The transient receptor potential channel TRPA1: from gene to pathophysiology. Pflugers Arch 464, 425–458 (2012). 23001121 10.1007/s00424-012-1158-z

[b14] Laing R. J. & Dhaka A. ThermoTRPs and Pain. Neuroscientist 22, 171–187 (2016). 25608689 10.1177/1073858414567884 PMC4510032

[b15] Ogawa N., Kurokawa T. & Mori Y. Sensing of redox status by TRP channels. Cell Calcium, (2016). 10.1016/j.ceca.2016.02.009 26969190

[b16] Gavva N. R. . Pharmacological blockade of the vanilloid receptor TRPV1 elicits marked hyperthermia in humans. Pain 136, 202–210 (2008). 18337008 10.1016/j.pain.2008.01.024

[b17] Caputo A. . TMEM16A, a membrane protein associated with calcium-dependent chloride channel activity. Science 322, 590–594 (2008). 18772398 10.1126/science.1163518

[b18] Yang Y. D. . TMEM16A confers receptor-activated calcium-dependent chloride conductance. Nature 455, 1210–1215 (2008). 18724360 10.1038/nature07313

[b19] Schroeder B. C., Cheng T., Jan Y. N. & Jan L. Y. Expression cloning of TMEM16A as a calcium-activated chloride channel subunit. Cell 134, 1019–1029 (2008). 18805094 10.1016/j.cell.2008.09.003 PMC2651354

[b20] Cho H. . The calcium-activated chloride channel anoctamin 1 acts as a heat sensor in nociceptive neurons. Nat Neurosci 15, 1015–1021 (2012). 22634729 10.1038/nn.3111

[b21] Lee B., Cho H., Jung J., Yang Y. D., Yang D. J. & Oh U. Anoctamin 1 contributes to inflammatory and nerve-injury induced hypersensitivity. Mol Pain 10, 5 (2014). 24450308 10.1186/1744-8069-10-5 PMC3929161

[b22] Jin X. . Activation of the Cl- channel ANO1 by localized calcium signals in nociceptive sensory neurons requires coupling with the IP3 receptor. Sci Signal 6, ra73 (2013). 10.1126/scisignal.2004184 PMC4135425 23982204

[b23] Jin X., Shah S., Du X., Zhang H. & Gamper N. Activation of Ca(2+) -activated Cl(−) channel ANO1 by localized Ca(2+) signals. J Physiol 594, 19–30 (2016). 25398532 10.1113/jphysiol.2014.275107 PMC4704509

[b24] Takayama Y., Uta D., Furue H. & Tominaga M. Pain-enhancing mechanism through interaction between TRPV1 and anoctamin 1 in sensory neurons. Proc Natl Acad Sci USA 112, 5213–5218 (2015). 25848051 10.1073/pnas.1421507112 PMC4413337

[b25] Sapio J. P., Sethachutkul K. & Moody J. E. Simultaneous GLC determination of methyl salicylate and menthol in a topical analgesic formulation. J Pharm Sci 68, 506–508 (1979). 438980 10.1002/jps.2600680431

[b26] Wright A. Oil of peppermint as a local anesthetic. Lancet 2, 726 (1870).

[b27] Peier A. M. . A TRP channel that senses cold stimuli and menthol. Cell 108, 705–715 (2002). 11893340 10.1016/s0092-8674(02)00652-9

[b28] Knowlton W. M. . A sensory-labeled line for cold: TRPM8-expressing sensory neurons define the cellular basis for cold, cold pain, and cooling-mediated analgesia. J Neurosci 33, 2837–2848 (2013). 23407943 10.1523/JNEUROSCI.1943-12.2013 PMC3711390

[b29] Proudfoot C. J. . Analgesia mediated by the TRPM8 cold receptor in chronic neuropathic pain. Curr Biol 16, 1591–1605 (2006). 16920620 10.1016/j.cub.2006.07.061

[b30] Xiao B., Dubin A. E., Bursulaya B., Viswanath V., Jegla T. J. & Patapoutian A. Identification of transmembrane domain 5 as a critical molecular determinant of menthol sensitivity in mammalian TRPA1 channels. J Neurosci 28, 9640–9651 (2008). 18815250 10.1523/JNEUROSCI.2772-08.2008 PMC2678945

[b31] Billen B. . Different ligands of the TRPV3 cation channel cause distinct conformational changes as revealed by intrinsic tryptophan fluorescence quenching. J Biol Chem 290, 12964–12974 (2015). 25829496 10.1074/jbc.M114.628925 PMC4432310

[b32] Takaishi M. . Reciprocal effects of capsaicin and menthol on thermosensation through regulated activities of TRPV1 and TRPM8. J Physiol Sci 66, 143–155 (2016). 26645885 10.1007/s12576-015-0427-y PMC4752590

[b33] Macpherson L. J., Hwang S. W., Miyamoto T., Dubin A. E., Patapoutian A. & Story G. M. More than cool: promiscuous relationships of menthol and other sensory compounds. Mol Cell Neurosci 32, 335–343 (2006). 16829128 10.1016/j.mcn.2006.05.005

[b34] Takayama Y., Shibasaki K., Suzuki Y., Yamanaka A. & Tominaga M. Modulation of water efflux through functional interaction between TRPV4 and TMEM16A/anoctamin 1. FASEB J 28, 2238–2248 (2014). 24509911 10.1096/fj.13-243436

[b35] Chun H. . Protons inhibit anoctamin 1 by competing with calcium. Cell Calcium 58, 431–441 (2015). 26183761 10.1016/j.ceca.2015.06.011

[b36] Bandell M. . High-throughput random mutagenesis screen reveals TRPM8 residues specifically required for activation by menthol. Nat Neurosci 9, 493–500 (2006). 16520735 10.1038/nn1665

[b37] Karashima Y. . Bimodal action of menthol on the transient receptor potential channel TRPA1. J Neurosci 27, 9874–9884 (2007). 17855602 10.1523/JNEUROSCI.2221-07.2007 PMC6672629

[b38] Nagatomo K. & Kubo Y. Caffeine activates mouse TRPA1 channels but suppresses human TRPA1 channels. Proc Natl Acad Sci USA 105, 17373–17378 (2008). 18988737 10.1073/pnas.0809769105 PMC2582301

[b39] Alessandri-Haber N. . Hypotonicity induces TRPV4-mediated nociception in rat. Neuron 39, 497–511 (2003). 12895423 10.1016/s0896-6273(03)00462-8

[b40] Alessandri-Haber N., Dina O. A., Yeh J. J., Parada C. A., Reichling D. B. & Levine J. D. Transient receptor potential vanilloid 4 is essential in chemotherapy-induced neuropathic pain in the rat. J Neurosci 24, 4444–4452 (2004). 15128858 10.1523/JNEUROSCI.0242-04.2004 PMC6729449

[b41] Alessandri-Haber N., Joseph E., Dina O. A., Liedtke W. & Levine J. D. TRPV4 mediates pain-related behavior induced by mild hypertonic stimuli in the presence of inflammatory mediator. Pain 118, 70–79 (2005). 16213085 10.1016/j.pain.2005.07.016

[b42] Grant A. D. . Protease-activated receptor 2 sensitizes the transient receptor potential vanilloid 4 ion channel to cause mechanical hyperalgesia in mice. J Physiol 578, 715–733 (2007). 17124270 10.1113/jphysiol.2006.121111 PMC2151332

[b43] Alessandri-Haber N., Dina O. A., Joseph E. K., Reichling D. B. & Levine J. D. Interaction of transient receptor potential vanilloid 4, integrin, and SRC tyrosine kinase in mechanical hyperalgesia. J Neurosci 28, 1046–1057 (2008). 18234883 10.1523/JNEUROSCI.4497-07.2008 PMC6671413

[b44] Chen Y. . TRPV4 is necessary for trigeminal irritant pain and functions as a cellular formalin receptor. Pain 155, 2662–2672 (2014). 25281928 10.1016/j.pain.2014.09.033 PMC4295825

[b45] Kanju P. . Small molecule dual-inhibitors of TRPV4 and TRPA1 for attenuation of inflammation and pain. Sci Rep 6, 26894 (2016). 27247148 10.1038/srep26894 PMC4887995

[b46] Huang F. . Studies on expression and function of the TMEM16A calcium-activated chloride channel. Proc Natl Acad Sci USA 106, 21413–21418 (2009). 19965375 10.1073/pnas.0911935106 PMC2781737

[b47] Huang F. . Calcium-activated chloride channel TMEM16A modulates mucin secretion and airway smooth muscle contraction. Proc Natl Acad Sci USA 109, 16354–16359 (2012). 22988107 10.1073/pnas.1214596109 PMC3479591

[b48] Scudieri P. . Association of TMEM16A chloride channel overexpression with airway goblet cell metaplasia. J Physiol 590, 6141–6155 (2012). 22988141 10.1113/jphysiol.2012.240838 PMC3530122

[b49] Xu Z., Lefevre G. M., Gavrilova O., Foster St Claire M. B., Riddick G. & Felsenfeld G. Mapping of long-range INS promoter interactions reveals a role for calcium-activated chloride channel ANO1 in insulin secretion. Proc Natl Acad Sci USA 111, 16760–16765 (2014). 25385647 10.1073/pnas.1419240111 PMC4250121

[b50] Crutzen R. . Anoctamin 1 (Ano1) is required for glucose-induced membrane potential oscillations and insulin secretion by murine beta-cells. Pflugers Arch (2015). 10.1007/s00424-015-1758-5 PMC4792454 26582426

[b51] Brunner J. D., Lim N. K., Schenck S., Duerst A. & Dutzler R. X-ray structure of a calcium-activated TMEM16 lipid scramblase. Nature 516, 207–212 (2014). 25383531 10.1038/nature13984

[b52] Manglik A. . Structure-based discovery of opioid analgesics with reduced side effects. Nature 537, 185–190 (2016). 27533032 10.1038/nature19112 PMC5161585

