# Supplementary material for: 4-isopropylcyclohexanol has potential analgesic effects through the inhibition of anoctamin 1, TRPV1 and TRPA1 channel activities
Source: Sci Rep. 2017 Feb 22;7:43132. doi: 10.1038/srep43132 (PMC5320485; doi:10.1038/srep43132)

**Title**

4-isopropylcyclohexanol has potential analgesic effects through the inhibition of anoctamin 1, TRPV1 and TRPA1 channel activities

**Authors and Affiliations**

Yasunori Takayama^1, 2, *^, Hidemasa Furue^2, 3^ and Makoto Tominaga^1, 2, 4 *^

^1^Division of Cell Signaling, Okazaki Institute for Integrative Bioscience (National Institute for Physiological Sciences), National Institutes of Natural Sciences, 5-1 Higashiyama, Myodaiji, Okazaki, Aichi, JAPAN 444-8787

^2^Department of Physiological Sciences, the Graduate University for Advanced Studies, 5-1 Higashiyama, Myodaiji, Okazaki, Aichi, JAPAN 444-8787

^3^Division of Neural Signaling, National Institute for Physiological Sciences, National Institutes of Natural Sciences, 5-1 Higashiyama, Myodaiji, Okazaki, Aichi, JAPAN 444-8787

^4^Institute for Environmental and Gender-Specific Medicine, Juntendo University

***corresponding authors**

Yasunori Takayama: 5-1 Higashiyama, Myodaiji, Okazaki, Aichi, JAPAN, +81-564-59-5287, takayama@nips.ac.jp

Makoto Tominaga: 5-1 Higashiyama, Myodaiji, Okazaki, Aichi, JAPAN, +81-564-59-5286, tomianga@nips.ac.jp

**Supplementary legends and figures**

**Supplementary Figure 1: ANO1 currents in several calcium concentrations.**

(**a**) A representative trace of ANO1-mediated currents in HEK293T cells at 300 nM intracellular free calcium. The pipette holding potential was 0 mV and 500 msec step pulses were applied between -100 and +100 mV with 20 mV increments. (**b**) Current-voltage relationships for ANO1-mediated currents at the beginning (left) and ending (right) of the step pulses indicated by arrowheads in (a).

**Supplementary Figure 2: Antagonistic effects of *l*-menthol on mTRPV1 currents.**

(**a**) A representative trace for capsaicin (100 nM)-induced mTRPV1-medited current that was inhibited by 3 mM *l*-menthol in HEK293T cells. The pipette holding potential was -60 mV and 500 msec ramp pulses from -100 to +100 mV were applied every 5 sec. (**b**) Averaged values for mTRPV1-mediated currents in the absence and presence of 3 mM *l*-menthol. *** *p* < 0.001, Student’s t-test.

**
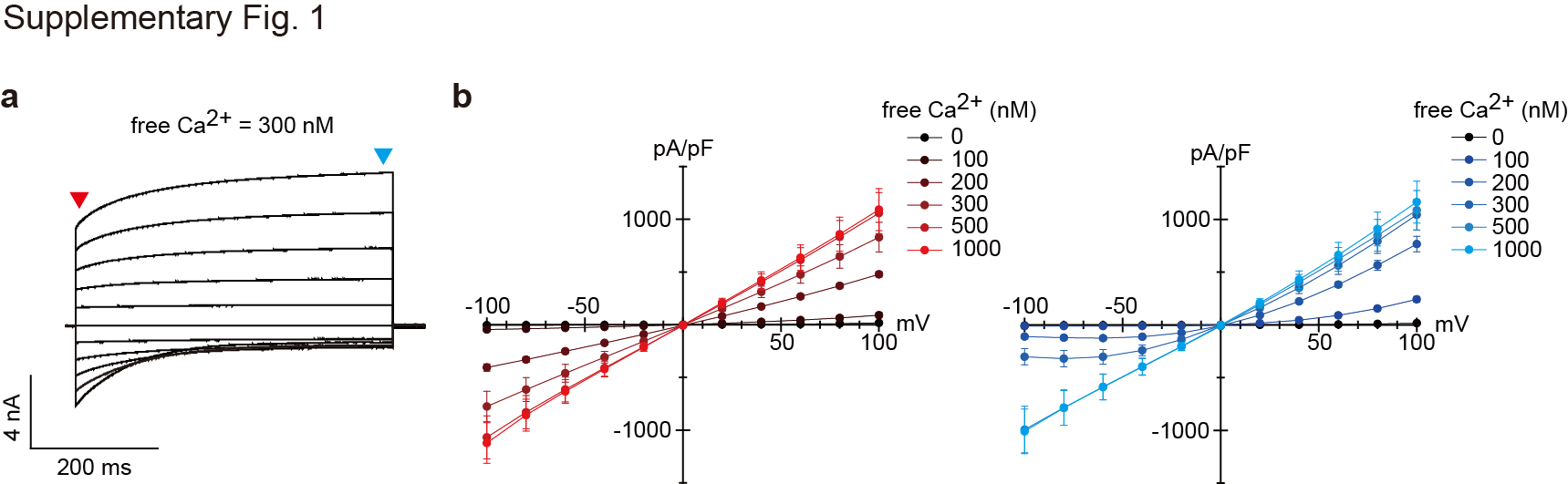
**


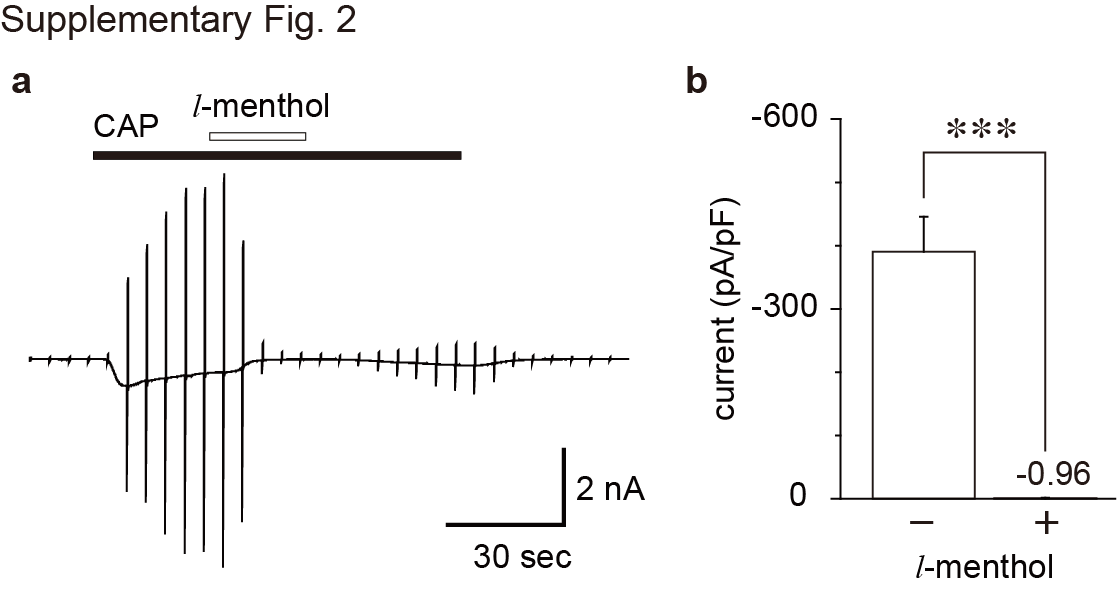

Supplement: Supplementary Information [file srep43132-s1.doc]
